# Receptor-Tyrosine-Kinase-Targeted Therapies for Head and Neck Cancer

**DOI:** 10.1155/2011/982879

**Published:** 2011-06-07

**Authors:** Lisa A. Elferink, Vicente A. Resto

**Affiliations:** ^1^Departments of Neuroscience and Cell Biology, University of Texas Medical Branch, Galveston, TX 77555-1074, USA; ^2^Department of Otolaryngology and UTMB Cancer Center, University of Texas Medical Branch, Galveston, TX 77555, USA

## Abstract

Molecular therapeutics for treating epidermal growth factor receptor-(EGFR-) expressing cancers are a specific method for treating cancers compared to general cell loss with standard cytotoxic therapeutics. However, the finding that resistance to such therapy is common in clinical trials now dampens the initial enthusiasm over this targeted treatment. Yet an improved molecular understanding of other receptor tyrosine kinases known to be active in cancer has revealed a rich network of cross-talk between receptor pathways with a key finding of common downstream signaling pathways. Such cross talk may represent a key mechanism for resistance to EGFR-directed therapy. Here we review the interplay between EGFR and Met and the type 1 insulin-like growth factor receptor (IGF-1R) tyrosine kinases, as well as their contribution to anti-EGFR therapeutic resistance in the context of squamous cell cancer of the head and neck, a tumor known to be primarily driven by EGFR-related oncogenic signals.

## 1. Introduction

Squamous cell carcinoma of the head and neck (HNSCC) is a heterogeneous disease that includes tumors arising from the mucosal epithelial surface of the oral cavity, oropharynx, hypopharynx, and larynx. Although these tumors originate within different anatomic sites within the upper aerodigestive tract, they are histologically identical (95% of HNSCC are squamous cell carcinomas), share common etiologic risk factors and overlapping metastatic target site profiles (reviewed in [1–3]). Recent genetic analysis of human head and neck tumors has revealed common molecular alterations including p53 mutation, p14ARF, and p16 methylation, as well as Cyclin D and EGFR amplification [3–6]. Despite these similarities, the distinct anatomic subsites are associated with differing rates of regional metastasis—for example, vocal cord lesions tend to metastasize less frequently than oropharyngeal or hypopharyngeal lesions. This variation may be attributed to differing densities of lymph draining vessels within each of the relevant subsites. Patients who exhibit metastases into the regional nodal basin exhibit a 50% decrease in survival irrespective of treatment [7–15]. The incidence of HNSCC has continued to increase over the last 3 decades. Currently, it is the 5th leading cause of cancer by incidence and the 6th leading cause of cancer mortality in the world [16, 17]. Recurrent and/or metastatic HNSCC patients have a poor prognosis, with a median survival of less than 1-2 years [18, 19].

Several lines of evidence indicate that cancer is a disease resulting from dynamic changes in the genome that promote the progressive transformation of normal human cells into highly malignant derivatives [20, 21]. During this process, cancer cells acquire several unique capabilities including self-sufficiency in response to growth signals, insensitivity to antigrowth signals, evasion of programmed death (apoptosis), limitless replicative potential, sustained angiogenesis as well as invasion and metastasis, reprogramming of energy metabolism, and avoiding immune destruction [21, 22]. Detailed global genomic analyses of several human tumors has revealed that certain classes of signaling proteins appear to be targeted more frequently by oncogenic mutations [23]. Receptor tyrosine kinases (RTKs) are a good example. Of the 59 transmembrane RTKs identified to date, dysregulation of ~30 RTKs are associated with neoplastic transformation and cancer progression [23–25]. Interestingly, ninety percent of primary head and neck squamous cell cancers, irrespective of subsite, have alterations in members of the epidermal growth factor (EGF) family of receptor tyrosine kinases (ErbBs), in particular ErbB1/EGFR [26]. Ten to fifteen percent of tumors will also have an alteration in another EGFR family member, the ErbB2/HER2/*Neu* receptor [27, 28]. These findings suggest a strong etiologic role for RTK dysregulation in this type of tumors. Given this association, patients with head and neck squamous cell cancers are well positioned to benefit from existing and future molecular targeted agents directed against oncogenic RTKs such as EGFR (reviewed in [29]). 

 RTKs are a family of transmembrane proteins that mediate many important physiological processes in both normal and cancerous cells. Ligand binding to the extracellular domain of RTKs induces receptor dimerization and activation of RTK activity. Subsequent autophosphorylation of the receptor at specific tyrosine residues within the cytoplasmic domain generates binding sites for proteins that relay downstream biological signals to regulate protein function, protein-protein interactions, and gene expression. Under physiological conditions, RTK signaling is temporally and spatially regulated. However, RTKs that become dysregulated can contribute to cellular transformation. RTK dysregulation can occur through several mechanisms including gene amplification or RTK overexpression, chromosomal translocation to produce constitutively active RTKs, gain of function mutations or deletions that promote ligand-independent RTK activity, escape from negative regulatory mechanisms or local environmental changes, all of which lead to potent oncogenic signaling and hence neoplastic growth. These complex signaling networks use multiple factors to drive the outcome of RTK signaling. Although often depicted as linear pathways, they actually represent an integrated network with various modes of cross-talk, overlapping and distinct functions. Known signaling pathways involved in head and neck tumorigenesis include the phosphatidylinositol-3-kinase (PI3K)-AKT-mammalian target of rapamycin (mTOR), signal transducer and activator of transcription (STATS) and Raf kinase-mitogen-activated protein kinase kinase (MEK)-p42/p44 mitogen activated protein kinase (MAPK) signaling pathways [1, 30]. This review highlights three RTK signaling pathways involved in head and neck squamous cell carcinoma; EGFR, the type 1 insulin-like growth factor receptor (IGF-1R) and the hepatocyte growth factor (HGF) receptor (Met). This short review will explore the relative contribution of each signaling axis to disease progression, potential modes of cross-talk, and targeted clinical approaches under investigation for disease management.

## 2. EGFR Amplification in Head and Neck Cancers

The EGFR family of RTKs is comprised of four different receptors known as ErbB1 (also referred to as EGFR), ErbB2 (HER2/*Neu* in rodents), ErbB3 (Her3), and ErbB4 (HER4) (reviewed in [31–33]). Each receptor, with the exception of ErbB3, contain an intracellular tyrosine kinase domain that is activated by binding to extracellular EGF-like ligands, which result in receptor dimerization and hence activation of downstream signaling cascades including MAPK, PI3K/AKT and Stat signaling. Eleven EGF-like ligands have been identified to date that can be categorized into four groups—those that bind EGFR only (EGF, Transforming Growth Factor alpha (TGF*α*), and amphiregulin), those that bind to EGFR and HER4 (heparin binding-EGF, betacellulin and epiregulin), those binding directly to either HER3 and HER4 (neuregulin 1 and neuregulin 2) and HER4 binding only (neuregulin 3 and neuregulin 4) (reviewed in [34]). Epigen, the most recently discovered member of the EGF-like ligand family appears to be a low affinity and broad specificity ligand that effectively activates EGFR [35]. Epigen is unable to activate HER3 and HER4 in the absence of ErbB2 expression. ErbB2 is considered a ligand-less coreceptor as it does not have any known ligands that bind directly with high affinity, despite its established role as a potent oncogene in several cancer types including breast, colorectal, nonsmall cell lung carcinoma (NSCLC) and HNSCC [36, 37]. 

Aberrant EGFR activity has been strongly linked to the etiology of 58–90% of HNSCC [26, 38]. These rates can vary due to the inclusion of cancers from different subsites within the head and neck, methods used to assess gene amplification and tumor scoring methods. In contrast to lung adenocarcinomas in which activating EGFR mutations result in ligand-independent signaling [39–43], such activating EGFR mutations are infrequent in HNSCC [44, 45]. EGFR gene amplification resulting in upwards of 12 copies per cell has been reported in HNSCC patients compared to copy numbers detected in normal mucosa from noncancer patients [46]. This and other pathways of ligand-independent receptor activation that do not require EGFR overexpression have been characterized as the likely drivers of EGFR activity in HNSCC. 

**Figure 1:**
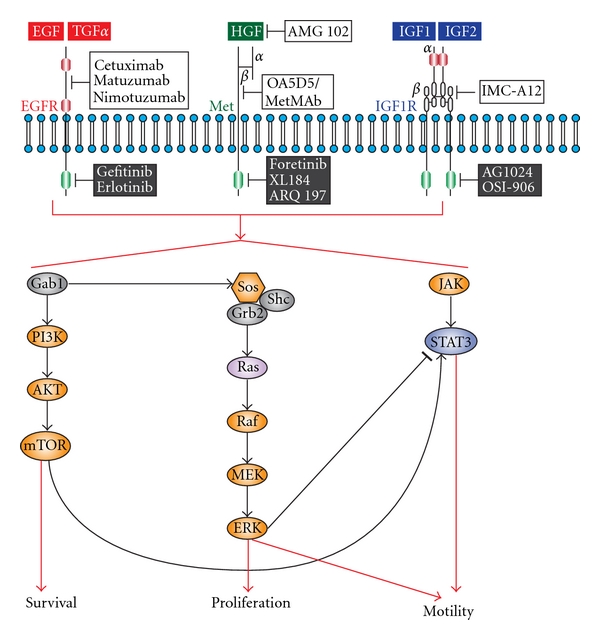
Targeted RTK and their signal transduction routes in head and neck cancer. The EGFR, Met, and IGF-1R receptors and their prototypic ligands are shown. Cysteine-rich domains (red box) and fibronectin type III-like domain (grey box) are indicated in the extracellular domains of the EGFR and IGF-1R, respectively. Cytoplasmic tyrosine kinase domains for each receptor are indicated (green boxes). The symbols *α* and *β* denote distinct RTK subunits. Targeted humanized monoclonal antibodies for extracellular components (white box) and TKIs (black box) for each receptor signaling axis is shown.

EGFR gene amplification remains a strong indicator for poor patient survival, radioresistance, and locoregional failure [47–49]. EGFR overexpression is detected in healthy mucosa in cancer patients (field cancerization) that will increase in proportion to observed histological abnormalities such as hyperplasia, carcinoma *in situ* and invasive carcinoma, indicating that it is an early event in HNSCC. Accordingly, significant effort has focused on EGFR signaling as a therapeutic target for treating HNSCC patients. The FDA has approved several EGFR-targeted reagents for treating HNSCC. Cetuximab, matuzumab and nimotuzumab represent humanized antiEGFR antibodies, whereas gefitinib and erlotinib are small tyrosine kinase inhibitors (TKIs) (Figure 1). Cetuximab (Erbitux) competitively inhibits endogenous ligand-binding to EGFR and thereby inhibits subsequent receptor activation [50–53]. Cetuximab is a valuable treatment option in head and neck patients as it synergizes with current treatment modalities. Cetuximab enhances the effects of many standard cytotoxic agents, including cisplatin (the conventional platinum-fluorouracil chemotherapeutic), and in combination with chemotherapy it can elicit antitumor responses in tumors that previously failed to respond to that chemotherapy [54]. Cetuximab has also been reported to enhance radiation-induced apoptosis. Notably, cetuximab did not dramatically exacerbate the common toxic effects associated with radiotherapy of the head and neck, including mucositis, xerostomia, dysphagia, pain, weight loss, and performance status deterioration [55]. Cetuximab has been approved for use in combination with radiation for treating patients with locally advanced HNSCC [56] and as monotherapy for patients with recurrent HNSCC [57]. Matuzumab (formerly EMD 72000) binds to EGFR with high specificity and affinity to block receptor signaling, and also modulates antibody-dependent cellular cytotoxicity (ADCC) when combined with cetuximab [58–60]. Phase I clinical trials report excellent antitumor activity of matuzumab against several human tumor types including head and neck cancers [61]. A randomized Phase IIb, four-arm, open-label study recently assessed the safety and efficacy of nimotuzumab in combination with radiation therapy (RT) or chemoradiation therapy (CRT) in patients with advanced (Stage III or IVa) HNSCC [62]. The addition of nimotuzumab to both the radiation and chemoradiation regimens was reported to improve the overall response rate, survival rate at 30 months, median progression-free survival and median overall survival. A combined group analysis of the nimotuzumab arms versus the non-nimotuzumab arms demonstrated a significant difference in overall survival favoring nimotuzumab. This study is compelling as patient response rates compare favorably with studies combining cetuximab with radiotherapy, but with fewer side effects [62]. Gefitinib (Iressa) is a small molecule TKI-targeted to the intracellular active site for phosphorylation that has been tested in clinical trials involving HNSCC patients, as a single agent or in combination with radiation treatment. Unfortunately, gefitinib has shown limited clinical efficacy with response rates of 10–15% [63, 64]. Erlotinib is a selective inhibitor of the EGFR that also shows antitumor activity in HNSCC comparable to standard combination chemotherapy [65].

## 3. Targeting IGF-1R Signaling in Head and Neck Cancers

Another promising RTK under preclinical and clinical evaluation for head and neck cancers includes the IGF-1R (reviewed in [66, 67]). Two ligands, insulin-like growth factor 1 (IGF1) and IGF2 bind to IGF-1R. Ligand binding to the IGF-1R stimulates its intrinsic tyrosine kinase activity, activating downstream signaling networks including Ras-Raf, MAPK and ERK, and PI3K (Figure 1) to drive cellular functions such as cell growth, survival and differentiation. It is widely accepted that the IGF-axis activates antiapoptotic signaling, which in turn upregulates the PI3K-Akt and MAPK pathways in cancer cells [68]. Additionally, IGF-IR also regulates vascular endothelial growth factor (VEGF) production, suggesting a role in tumor angiogenesis [69]. Several studies indicate that IGF-1R is overexpressed and functional in 94% of HNSCC patient samples [70, 71]. Consistent with this, IGF-IR signaling significantly enhances the proliferation, motility and tumorigenicity of human head and neck cancer cell lines [71]. IGF-1R down regulation in a HNSCC cell line using antisense oligonucleotides resulted in a dose-dependent decrease in cellular proliferation, induction of apoptosis, caspase activation and reduced expression of proangiogenic cytokines such as VEGF. Interest in targeting the IGF-1R in HNSCC was bolstered by the observation that treatment of head and neck cancer cells with either IGF or EGF resulted in IGF-IR and EGFR heterodimerization [71, 72]. However, only IGF resulted in the phosphorylation of both receptors. Using a mouse xenograft model for HNSCC, treatment with antibodies against IGF-1R, EGFR or both receptors resulted in significant differences in median tumor volume. It remains to be determined whether cellular cross-talk between IGF-1R and EGFR has an important role in determining the biological aggressiveness of HNSCC or resistance to EGFR-targeted therapies. 

Several monoclonal antibodies and TKIs for IGF-1R have been tested in preclinical studies and early phase clinical studies. However, the efficacy of IGF-1R-targeted therapy for treating patients with HNSCC, particularly cross-talk with EGFR, warrants further investigation. To date, the effect of blocking oncogenic IGF-1R and EGFR signaling have been studied more extensively in breast cancer cell lines [73–75]. Treatment with gefitinib and AG1024, a TKI for IGF-1R reduced cell proliferation when used as single agents and showed an additive effect when used in combination [76, 77]. Targeting IGF-1R and EGFR signaling is currently under evaluation in hormone-sensitive metastatic breast cancer using the IGF-1R inhibitor OSI-906 and the EGFR TKI erlotinib, although results are not yet available (http://www.clinicaltrials.gov/, Identifier NCT01205685). Similarly, an exploratory study to assess the modulation of biomarkers in HNSCC patients treated preoperatively with cetuximab and/or IMC-A12, a humanized antiIGF-1R monoclonal antibody is currently underway (http://www.clinicaltrials.gov/, Identifier NCT00617734). These studies will be critical for evaluating whether the use of anti-IGF-1R and EGFR-targeted treatments will be more effective than single-agent modalities for treating patients with HNSCC.

## 4. A Role for Met/HGF Signaling in Head and Neck Cancers

The Met/HGF signaling axis is frequently upregulated and functional in HNSCC. The Met receptor is a single pass transmembrane protein that upon binding its ligand HGF—also known as scatter factor-promotes increased cell proliferation, survival and motility (reviewed in [78, 79]). HGF is the only physiological ligand for Met and is secreted as an inactive precursor polypeptide chain by mesenchymal cells. HGF is proteolytically cleaved to form an active *α/β* heterodimer by a number of serine proteases including urokinase plasminogen activator (uPA), tissue-type plasminogen activator (tPA), coagulation factors X. XI and XII. Met is a disulphide-linked *α/β* heterodimer derived from the proteolytic cleavage of a 170 KDa precursor. The *α* chain is exclusively extracellular while the *β* chain spans the membrane once. The *α* chain and N-terminal region of the *β*-chain form *α* sema domain, a seven *β*-propeller structure in which blades 2 and 3 bind to HGF. The sema domain is flanked by a cysteine-rich region followed by four immunoglobulin repeats. It is proposed that the cysteine-rich region and immunoglobulin repeat domains undergo a conformational change following HGF binding allowing for Met dimerization [80, 81].

Binding of HGF to Met results in receptor autophosphorylation at key catalytic residues and subsequent recruitment of several cytosolic signaling molecules that are shared with the EGFR and IGF-1R signaling pathways, including the Grb2/Sos complex, the p85 regulatory subunit of PI3K, Gab1 and Jak/Stat3 (Figure 1). Subsequent activation of the MAPK and Jun-N-terminal Kinase (JNK) pathways is responsible for the mitogenic and motogenic properties of Met/HGF signaling resulting in “invasive growth”, depending on the physiological setting [79].

Increased Met signaling in human cancers can be the result of enhanced ligand-binding (autocrine and paracrine), Met overexpression or missense mutations that often induce constitutive kinase activity, failure of Met down regulation and interactions with other cell surface receptors such as EGFR (reviewed in [82–84]). Met is overexpressed in 84% of HNSCC patient samples [85]. Interestingly, amplification of the MET gene (>10 copies per cell) is present only in 3 of 23 (13%) tumor tissues. HGF overexpression is detected in 45% of HNSCCs, suggesting that HGF functions predominantly in a paracrine manner to drive Met signaling in these cancers. Moreover, high levels of HGF are detected in HNSCC patient plasma samples [86] supporting the idea that ligand availability is not a limiting factor for Met activation. Mutations in the Met ligand-binding domain (T230M/E168D), transmembrane or JM domain (R988C, T1010I) and the tyrosine kinase domain (T1275I, V14333I) have also been identified in HNSCC tumor samples [85], although their relative contribution to HNSCC progression remains to be determined. Two somatic Met mutations have been detected in HNSCC that result in constitutively active receptor signaling that confers an invasive phenotype when ectopically expressed in cell lines [87]. The Y1230C mutation confers anchorage-independent growth and an invasive phenotype in transfected cells, whereas the Y1235D Met mutation stimulates epithelial cells to invade reconstituted basement membrane in the absence of HGF. In the case of the MetY1235D mutation, genomic analyses of HNSCC patient samples detected the presence of this mutant allele in 50% of metastatic tumors versus 2–6% in primary tumors, raising the possibility that this could be a critical genetic lesion for the acquisition of a metastatic phenotype. Alternatively, increased Met signaling could afford HNSCC a selective advantage for growth and/or survival in metastatic sites, such as the lymph node and lung. Indeed several studies indicate that Met overexpression correlates highly with lymph node metastasis, pathologic stage, and disease reoccurrence [88–91]. Moreover, patient survival was significantly reduced in biopsy samples with positive Met expression relative to negative Met expression, suggesting the association of Met with HNSCC disease progression. Consistent with these findings, treatment with the TKI PF-2341066 caused a significant reduction in tumor growth, a high level of apoptosis and cellular debris within the tumor using a xenograft animal model for HNSCC [91].

Selective inhibitors of Met/HGF signaling include humanized monoclonal antibodies for HGF and Met, and small-molecule tyrosine kinase inhibitors directed against Met (Figure 1). Although their efficacy for treating a variety of solid tumors is increasingly recognized, we await results of preclinical and clinical trials for head and neck cancer that are ongoing. The humanized antibody AMG 102 shows high potency towards the mature and processed form of HGF with no detected effects on proteolytic activation of proHGF. AMG 102 interferes with Met signaling, by competing with HGF for binding to the *β* chain of the Met receptor [92]. In phase I clinical studies in patients with advanced solid tumors, 70% of patients had a best response in terms of achieving stable disease [93, 94]. Drug-related toxicities included mild fatigue and gastrointestinal symptoms. Importantly, no antiAMG 102 antibodies were detected and circulating HGF levels were dose dependent [93]. Another promising clinical therapeutic is the one-armed 5D5 humanized antibody (OA5D5/MetMAb) directed against Met. MetMAb binds Met with high affinity, preventing HGF binding, Met phosphorylation, receptor internalization and downstream signaling events and has been shown to inhibit tumor growth in animal models by more than 95% [95, 96]. MetMAb is currently in phase I/II human clinical trials in comparison with erlotinib in patients with NSCLC (http://www.clinicaltrials.gov/, Identifier NCT00854308). Future clinical trials will be required to determine the suitability of AMG102 and MetMAb as either single agents or combinatorial therapeutics for treating HNSCC patients. Foretinib (formerly XL880) is a TKI whose primary targets include Met and VEGF, and to a lesser extent the platelet-derived growth factor (PDGF) receptor, Ron, Kit and TIE2 RTKs [97]. Foretinib recently completed phase II clinical trials in head and neck patients (http://www.clinicaltrials.gov/, Identifier NCT00725764). Interim results suggest that after 12 months, 12 of 18 patients had stable disease [83]. XL184 is another TKI agent entering phase III clinical trials. XL184 targets Met, VEGFR2, and Ret. A phase I dose-escalation study of the safety and pharmacokinetics of XL184 administered orally to patients with advanced malignancies (showed that, on average, patients survived for more than 3 months with several up to 6 months while on treatment) (reviewed in [84]). Due to encouraging data from this study, a randomized phase III trial of XL184 in HNSCC patients was initiated to investigate XL184 as a first-line treatment (compared with placebo) for survival benefit to patients with HNSCC (http://www.clinicaltrials.gov/, Identifier NCT00704730). ARQ197 (ArQule) is a nonATP-site competitive, selective small molecule inhibitor of the Met intracellular region [98]. Although the mechanism of ARQ197 is presently unknown, the results of phase I trials suggest potential antiinvasive activity for this compound [99]. Overall, Met, and HGF-targeted therapies have been well tolerated in clinical trials with negligible toxicities. However, it remains to be determined whether Met is a better therapeutic target than HGF. Clearly, in patients where Met is activated by autocrine HGF secretion, both HGF and Met targeted therapies may prove to be more efficacious treatment options.

## 5. Understanding Resistance to EGFR-Targeted Therapies in HNSCC

Acquired resistance is likely the result of several mechanisms including (1) EGFR mutations initially present as well as those acquired during therapy, (2) receptor independent activation of downstream signaling cascades, (3) cross-talk with other RTKs and converging signaling pathways and (4) environmental factors including inflammatory agents and viral infection. Resistance to cetuximab has been associated with the coexpression of the truncated EGFR mutant, EGFRvIII with wild-type EGFR. EGFRvIII is the result of an in frame deletion of exons 2–7 spanning the extracellular ligand-binding domain. The deletion results in a truncated EGFR receptor that signals in a ligand-independent manner [100]. EGFRvIII expression has been detected in 42% of HNSCC patient samples, and closely correlates with increased HNSCC cell proliferation *in vitro* and increased tumor growth using *in vivo *xenograft models. EGFRvIII preferentially activates the PI3K pathway instead of the Ras/Raf/MEK pathway, which is activated by wild-type EGFR [101]. Of particular interest to the therapeutic treatment of HNSCC, EGFRvIII expression decreases the proliferative response of EGFR expressing tumor cells to cetuximab treatment relative to vector control cells. In a recent study, EGFRvIII cells were shown to be resistant to the antiinvasive effects of cetuximab due to an increase in phosphorylation of STAT3 rather than increased PI3K signaling. EGF-induced expression of the STAT3 target gene HIF1*α* was abolished by cetuximab in HNSCC cells expressing wild-type EGFR under hypoxic conditions, but not in EGFRvIII-expressing HNSCC cells [102, 103]. These data suggest a role for EGFRvIII in mediating HNSCC resistance to cetuximab. 

Despite EGFRs critical role in the development of HNSCC, clinical data indicate modest clinical benefits for locoregional control and survival of head and neck cancer patients treated with EGFR-targeted therapies. HNSCC patients resistant to cetuximab, often succumb to local tumor recurrence as well as regional and distant metastasis. The addition of cetuximab to radiation therapy was reported to show improved locoregional disease control, progression-free survival, and overall survival in patients with locally advanced HNSCC [56]. However the data revealed a disproportionate benefit of cetuximab with radiotherapy to oropharyngeal cancer patients when compared to patients treated with hyperfractionated radiotherapy [57]. Accumulating evidence suggests that human papilloma virus (hpv) 16 status (Hpv+) is an important prognostic factor associated with a favorable outcome in a subset of head and neck cancers, including oropharyngeal and tonsilar cancers [104]. Hpv+ tumors tend to have unique genetic aberrations including decreased EGFR expression, whereas increased IGF-1R levels characteristic of HNSCC appear to be independent of hpv status. Clinically, hpv+ tumors are characterized by more favorable patient prognosis regarding disease-free survival as well as overall survival [104, 105], possibly as a result of increased genomic stability associated with global gene hypermethylation in hpv+ tumors [106]. Thus it will be interesting to determine whether hpv+ status explains some of the benefits derived from the addition of cetuximab to radiotherapy in this subset of HNSCC patients. At present, there are few clinical indicators of which HNSCC patients will most likely respond to EGFR-targeted therapies. Accordingly, strategies to optimize EGFR-targeted therapy remain an active area of research.

Additional mechanisms that result in EGFR activation include activating mutations in downstream signaling components or cross-talk between different RTK pathways. Activating mutations in the PI3KA oncogene occurs in 10% of HNSCC tumors [107] whereas elevated levels of phosphorylated STAT3 correlates with lymph node metastasis and poor patient prognosis [108–110]. Conversely, H-Ras mutations are infrequent in HNSCC cases (less than 5%), although a higher incidence has been detected in Asian populations and correlates with Areca nut chewing [111, 112].

Met signaling has been shown to contribute to resistance in cell lines derived from multiple tumor types including breast, gastric and lung. In one key study, NSCLC with activating mutations in the EGFR acquire resistance to the TKI gefitinib and erlotinib, by amplification of the Met gene to maintain Akt and Her3 signaling [113]. These studies underscore the role of cross-talk between RTKs to preferentially signal through the PI3K-Akt survival pathway as a mechanism for acquired drug resistance. The relevance of Met as a mechanism for escape from EGFR-targeted therapy in head and neck cancers remains to be determined. Hypoxia results in the transcriptional upregulation of Met gene expression via HIF1*α* in a number of tumors including head and neck [114], often downstream of EGFR signaling [115]. In normoxia, hydroxylation of 2 prolines in HIF1*α* enables its binding to the von Hippel-Lindau tumor suppressor protein (pVHL) linking HIF1*α* to a ubiquitin ligase complex. During hypoxia, minimal or no hydroxylation occurs enabling HIF1*α* to avoid proteasomal degradation and dimerize to other HIF family members such as HIF1  *β* and coactivators, to form an active transcriptional HIF complex on the hypoxia response element (HRE) of target genes such as MET [116]. The ubiquitin ligase catalyzes polyubiquitination of HIF1*α* targeting it for proteasomal degradation [117]. Under hypoxic conditions, increased Met signaling directs the invasive growth program, enabling cells to invade more oxygenated tissues [118]. Since Met has been reported to promote invasive and angiogenic effects in the tumor microenvironment, the use of HGF/Met inhibitors may afford a means of impairing tissue colonization as well as tumor vascularization in head and neck cancer patients. 

 Studies on other solid tumor types, most notably glioblastoma, indicate a role for IGF-1R upregulation in resistance to EGFR-targeted therapies [73]. IGF-1R mediates resistance to anti-EGFR therapy in primary glioblastoma through the continued activation of the PI3K/AKT survival pathway [119]. The apparent cooperation between IGF-1R and EGFR in promoting HNSCC pathogenesis as well as resistance to EGFR-targeted therapy, suggests an advantage to cotargeting these signaling axes for the treatment of head and neck cancers. To date, the effect of blocking oncogenic IGF-1R and EGFR signaling have been studied more extensively in breast cancer lines. Treatment with gefitinib and AG1024, a TKI for IGF-1R reduced cell proliferation when used as single agents and showed an additive effect when used in combination [76, 77]. Targeting IGF-1R and EGFR signaling is currently under evaluation in hormone-sensitive metastatic breast cancer using the IGF-1R inhibitor OSI-906 and the EGFR TKI erlotinib, although results are not yet available (http://www.clinicaltrials.gov/, Identifier NCT01205685). Similarly, an exploratory study to assess the modulation of biomarkers in HNSCC patients treated preoperatively with cetuximab and/or IMC-A12, a humanized antiIGF-1R monoclonal antibody is currently underway (http://www.clinicaltrials.gov/, Identifier NCT00617734). These studies will be critical for evaluating whether the use of antiIGF-1R and EGFR-targeted treatments will be more effective than single-agent modalities for treating patients with HNSCC.

## 6. Conclusions

Targeted therapies that block EGFR, Met, and IGF-1R signaling in head and neck cancers continue to show promising results in preclinical studies and clinical trials. However, it is difficult to predict which patients are most likely to benefit from these therapeutics and potential side effects during long-term *in vivo* use. Given the interplay between these RTK signaling pathways and the mediocre results obtained with monotherapy regimens thus far, clinical trials will be required to determine how EGFR-, Met-, and IGF-1R-targeted therapies can be used in combination in order to definitively abrogate their common downstream oncogenic signaling networks. Although gaps in our knowledge concerning the role of Met and IGF-1R in head and neck tumorigenesis, as well as acquired resistance to antiEGFR therapies remain to be addressed, efforts to translate current information towards clinical applications continue to be impressive.

## Abbreviations

EGF: Epidermal growth factor
EGFR: EGF receptor
ErbBs: Epidermal growth factor receptor family of receptor tyrosine kinases
HGF: Hepatocyte growth factor
HIF1*α*: Hypoxia-inducible factor 1 alpha subunit
HNSCC: Squamous cell carcinoma of the head and neck
Hpv: Human papilloma virus
HRE: Hypoxia response element
IGF: Insulin-like growth factor
IGF-1R: Type 1 insulin-like growth factor receptor
mTOR: Mammalian target of rapamycin
MAPK: p42/p44 Mitogen Activated Protein Kinase
Mek: MAPK kinase
Met: HGF receptor
NSCLC: Nonsmall cell lung carcinoma
PI3K: Phosphatidylinositol-3-kinase
PDGF: Platelet-derived growth factor
RTK: Receptor tyrosine kinase
STAT: Signal transducer and activator of transcription
TKI: Tyrosine kinase inhibitor
VEGF: Vascular endothelial growth factor
VEGFR: VEGF receptor
uPA: Urokinase plasminogen activator
tPA: Tissue-type plasminogen activator.

## References

[1] Molinolo AA, Amornphimoltham P, Squarize CH, Castilho RM, Patel V, Gutkind JS (2009). Dysregulated molecular networks in head and neck carcinogenesis. *Oral Oncology*.

[2] Bernier J, Bentzen SM, Vermorken JB (2009). Molecular therapy in head and neck oncology. *Nature Reviews Clinical Oncology*.

[3] Leemans CR, Braakhuis BJM, Brakenhoff RH (2011). The molecular biology of head and neck cancer. *Nature Reviews Cancer*.

[4] Chung CH, Parker JS, Karaca G (2004). Molecular classification of head and neck squamous cell carcinomas using patterns of gene expression. *Cancer Cell*.

[5] Bleijerveld OB, Brakenhoff RH, Schaaij-Visser TBM (2011). Protein signatures associated with tumor cell dissemination in head and neck cancer. *Journal of Proteomics*.

[6] Choi P, Jordan CD, Mendez E (2008). Examination of oral cancer biomarkers by tissue microarray analysis. *Archives of Otolaryngology—Head and Neck Surgery*.

[7] Forastiere A, Koch W, Trotti A, Sidransky D (2001). Head and neck cancer. *The New England Journal of Medicine*.

[8] Goldson TM, Han Y, Knight KB, Weiss HL, Resto VA (2010). Clinicopathological predictors of lymphatic metastasis in HNSCC: implications for molecular mechanisms of metastatic disease. *Journal of Experimental Therapeutics and Oncology*.

[9] Chan SW, Mukesh BN, Sizeland A (2003). Treatment outcome of N3 nodal head and neck squamous cell carcinoma. *Otolaryngology—Head and Neck Surgery*.

[10] Spector JG, Sessions DG, Haughey BH (2001). Delayed regional metastases, distant metastases, and second primary malignancies in squamous cell carcinomas of the larynx and hypopharynx. *Laryngoscope*.

[11] Klotch DW, Muro-Cacho C, Gal TJ (2000). Factors affecting survival for floor-of-mouth carcinoma. *Otolaryngology—Head and Neck Surgery*.

[12] Olsen KD, Caruso M, Foote RL (1994). Primary head and neck cancer: histopathologic predictors of recurrence after neck dissection in patients with lymph node involvement. *Archives of Otolaryngology—Head and Neck Surgery*.

[13] Sessions DG, Lenox J, Spector GJ, Chao C, Chaudry OA (2003). Analysis of treatment results for base of tongue cancer. *Laryngoscope*.

[14] Sessions DG, Spector GJ, Lenox J, Haughey B, Chao C, Marks J (2002). Analysis of treatment results for oral tongue cancer. *Laryngoscope*.

[15] Sessions DG, Spector GJ, Lenox J (2000). Analysis of treatment results for floor-of-mouth cancer. *Laryngoscope*.

[16] Shiboski CH, Schmidt BL, Jordan RCK (2005). Tongue and tonsil carcinoma: increasing trends in the U.S. population ages 20–44 years. *Cancer*.

[17] Jemal A, Siegel R, Xu J, Ward E (2010). Cancer statistics, 2010. *CA Cancer Journal for Clinicians*.

[18] Davies L, Welch HG (2006). Epidemiology of head and neck cancer in the United States. *Otolaryngology—Head and Neck Surgery*.

[19] Carvalho AL, Nishimoto IN, Califano JA, Kowalski LP (2005). Trends in incidence and prognosis for head and neck cancer in the United States: a site-specific analysis of the SEER database. *International Journal of Cancer*.

[20] Poage GM, Christensen BC, Houseman EA (2010). Genetic and epigenetic somatic alterations in head and neck squamous cell carcinomas are globally coordinated but not locally targeted. *PLoS One*.

[21] Hanahan D, Weinberg RA (2011). Hallmarks of cancer: the next generation. *Cell*.

[22] Hanahan D, Weinberg RA (2000). The hallmarks of cancer. *Cell*.

[23] Blume-Jensen P, Hunter T (2001). Oncogenic kinase signalling. *Nature*.

[24] Hunter T (2009). Tyrosine phosphorylation: thirty years and counting. *Current Opinion in Cell Biology*.

[25] Manning G, Whyte DB, Martinez R, Hunter T, Sudarsanam S (2002). The protein kinase complement of the human genome. *Science*.

[26] Grandis JR, Tweardy DJ (1993). Elevated levels of transforming growth factor *α* and epidermal growth factor receptor messenger RNA are early markers of carcinogenesis in head and neck cancer. *Cancer Research*.

[27] Cavalot A, Martone T, Roggero N, Brondino G, Pagano M, Cortesina G (2007). Prognostic impact of HER-2/neu expression on squamous head and neck carcinomas. *Head and Neck*.

[28] Rautava J, Jee KJ, Miettinen PJ (2008). ERBB receptors in developing, dysplastic and malignant oral epithelia. *Oral Oncology*.

[29] Fung C, Grandis JR (2010). Emerging drugs to treat squamous cell carcinomas of the head and neck. *Expert Opinion on Emerging Drugs*.

[30] Klein JD, Grandis JR (2010). The molecular pathogenesis of head and neck cancer. *Cancer Biology and Therapy*.

[31] Bae JH, Schlessinger J (2010). Asymmetric tyrosine kinase arrangements in activation or autophosphorylation of receptor tyrosine kinases. *Molecules and Cells*.

[32] Burgess AW (2008). EGFR family: structure physiology signalling and therapeutic target. *Growth Factors*.

[33] Jorissen RN, Walker F, Pouliot N, Garrett TPJ, Ward CW, Burgess AW (2003). Epidermal growth factor receptor: mechanisms of activation and signalling. *Experimental Cell Research*.

[34] Hynes NE, Lane HA (2005). ERBB receptors and cancer: the complexity of targeted inhibitors. *Nature Reviews Cancer*.

[35] Strachan L, Murison JG, Prestidge RL, Sleeman MA, Watson JD, Kumble KD (2001). Cloning and biological activity of epigen, a novel member of the epidermal growth factor superfamily. *The Journal of Biological Chemistry*.

[36] Yarden Y, Sliwkowski MX (2001). Untangling the ErbB signalling network. *Nature Reviews Molecular Cell Biology*.

[37] Kochupurakkal BS, Harari D, Di-Segni A (2005). Epigen, the last ligand of ErbB receptors, reveals intricate relationships between affinity and mitogenicity. *The Journal of Biological Chemistry*.

[38] Grandis JR, Tweardy DJ (1993). TGF-*α* and EGFR in head and neck cancer. *Journal of Cellular Biochemistry*.

[39] Lynch TJ, Bell DW, Sordella R (2004). Activating mutations in the epidermal growth factor receptor underlying responsiveness of non-small-cell lung cancer to gefitinib. *The New England Journal of Medicine*.

[40] Cortes-Funes H, Gomez C, Rosell R (2005). Epidermal growth factor receptor activating mutations in Spanish gefitinib-treated non-small-cell lung cancer patients. *Annals of Oncology*.

[41] Riely GJ, Pao W, Pham D (2006). Clinical course of patients with non-small cell lung cancer and epidermal growth factor receptor exon 19 and exon 21 mutations treated with gefitinib or erlotinib. *Clinical Cancer Research*.

[42] Paez JG, Jänne PA, Lee JC (2004). EGFR mutations in lung, cancer: correlation with clinical response to gefitinib therapy. *Science*.

[43] Pao W, Miller V, Zakowski M (2004). EGF receptor gene mutations are common in lung cancers from “never smokers” and are associated with sensitivity of tumors to gefitinib and erlotinib. *Proceedings of the National Academy of Sciences of the United States of America*.

[44] Willmore-Payne C, Holden JA, Layfield LJ (2006). Detection of epidermal growth factor receptor and human epidermal growth factor receptor 2 activating mutations in lung adenocarcinoma by high-resolution melting amplicon analysis: correlation with gene copy number, protein expression, and hormone receptor expression. *Human Pathology*.

[45] Jong WL, Young HS, Su YK (2005). Somatic mutations of EGFR gene in squamous cell carcinoma of the head and neck. *Clinical Cancer Research*.

[46] Temam S, Kawaguchi H, El-Naggar AK (2007). Epidermal growth factor receptor copy number alterations correlate with poor clinical outcome in patients with head and neck squamous cancer. *Journal of Clinical Oncology*.

[47] Ang KK, Berkey BA, Tu X (2002). Impact of epidermal growth factor receptor expression on survival and pattern of relapse in patients with advanced head and neck carcinoma. *Cancer Research*.

[48] Gupta AK, McKenna WG, Weber CN (2002). Local recurrence in head and neck cancer: relationship to radiation resistance and signal transduction. *Clinical Cancer Research*.

[49] Bentzen SM, Atasoy BM, Daley FM (2005). Epidermal growth factor receptor expression in pretreatment biopsies from head and neck squamous cell carcinoma as a predictive factor for a benefit from accelerated radiation therapy in a randomized controlled trial. *Journal of Clinical Oncology*.

[50] Sato JD, Kawamoto T, Le AD, Mendelsohn J, Polikoff J, Sato GH (1983). Biological effects in vitro of monoclonal antibodies to human epidermal growth factor receptors. *Molecular Biology & Medicine*.

[51] Kawamoto T, Sato JD, Le A (1983). Growth stimulation of A431 cells by epidermal growth factor: identification of high-affinity receptors for epidermal growth factor by an anti-receptor monoclonal antibody. *Proceedings of the National Academy of Sciences of the United States of America*.

[52] Gill GN, Kawamoto T, Cochet C (1984). Monoclonal anti-epidermal growth factor receptor antibodies which are inhibitors of epidermal growth factor binding and antagonists of epidermal growth factor-stimulated tyrosine protein kinase activity. *The Journal of Biological Chemistry*.

[53] Masui H, Kawamoto T, Sato JD (1984). Growth inhibition of human tumor cells in athymic mice by anti-epidermal growth factor receptor monoclonal antibodies. *Cancer Research*.

[54] Koutcher L, Sherman E, Fury M Concurrent cisplatin and radiation versus cetuximab and radiation for locally advanced head-and-neck cancer.

[55] Dequanter D, Shahla M, Paulus P, Lothaire P (2010). Cetuximab in the treatment of head and neck cancer: preliminary results outside clinical trials. *Cancer Management and Research*.

[56] Bonner JA, Harari PM, Giralt J (2006). Radiotherapy plus cetuximab for squamous-cell carcinoma of the head and neck. *The New England Journal of Medicine*.

[57] Cripps C, Winquist E, Devries MC, Stys-Norman D, Gilbert R (2010). Epidermal growth factor receptor targeted therapy in stages III and IV head and neck cancer. *Current Oncology*.

[58] Taylor RJ, Chan SL, Wood A (2009). Fc*γ*RIIIa polymorphisms and cetuximab induced cytotoxicity in squamous cell carcinoma of the head and neck. *Cancer Immunology, Immunotherapy*.

[59] Dechant M, Weisner W, Berger S (2008). Complement-dependent tumor cell lysis triggered by combinations of epidermal growth factor receptor antibodies. *Cancer Research*.

[60] Schmiedel J, Blaukat A, Li S, Knöchel T, Ferguson KM (2008). Matuzumab binding to EGFR prevents the conformational rearrangement required for dimerization. *Cancer Cell*.

[61] Vanhoefer U, Tewes M, Rojo F (2004). Phase I study of the humanized antiepidermal growth factor receptor monoclonal antibody EMD72000 in patients with advanced solid tumors that express the epidermal growth factor receptor. *Journal of Clinical Oncology*.

[62] Rodríguez MO, Rivero TC, Bahi RDC (2010). Nimotuzumab plus radiotherapy for unresectable squamous-cell carcinoma of the head and neck. *Cancer Biology and Therapy*.

[63] Ranson M, Hammond LA, Ferry D (2002). ZD1839, a selective oral epidermal growth factor receptor-tyrosine kinase inhibitor, is well tolerated and active in patients with solid, malignant tumors: results of a phase I trial. *Journal of Clinical Oncology*.

[64] Baselga J, Rischin D, Ranson M (2002). Phase I safety, pharmacokinetic, and pharmacodynamic trial of ZD1839, a selective oral epidermal growth factor receptor tyrosine kinase inhibitor, in patients with five selected solid tumor types. *Journal of Clinical Oncology*.

[65] Siu LL, Soulieres D, Chen EX (2007). Phase I/II trial of erlotinib and cisplatin in patients with recurrent or metastatic squamous cell carcinoma of the head and neck: a Princess Margaret Hospital Phase II Consortium and National Cancer Institute of Canada Clinical Trials Group study. *Journal of Clinical Oncology*.

[66] Pollak M (2008). Insulin and insulin-like growth factor signalling in neoplasia. *Nature Reviews Cancer*.

[67] Tao Y, Pinzi V, Bourhis J, Deutsch E (2007). Mechanisms of Disease: signaling of the insulin-like growth factor 1 receptor pathway—therapeutic perspectives in cancer. *Nature Clinical Practice Oncology*.

[68] Kurihara S, Hakuno F, Takahashi SI (2000). Insulin-like growth factor-I-dependent signal transduction pathways leading to the induction of cell growth and differentiation of human neuroblastoma cell line SH-SY5Y: the roles of MAP kinase pathway and PI 3-kinase pathway. *Endocrine Journal*.

[69] Tang Y, Zhang D, Fallavollita L, Brodt P (2003). Vascular endothelial growth factor C expression and lymph node metastasis are regulated by the type I insulin-like growth factor receptor. *Cancer Research*.

[70] Friedrich RE, Hagel C, Bartel-Friedrich S (2010). Insulin-like growth factor-1 receptor (IGF-1R) in primary and metastatic undifferentiated carcinoma of the head and neck: a possible target of immunotherapy. *Anticancer Research*.

[71] Barnes CJ, Ohshiro K, Rayala SK, El-Naggar AK, Kumar R (2007). Insulin-like growth factor receptor as a therapeutic target in head and neck cancer. *Clinical Cancer Research*.

[72] Liu S, Jin F, Dai W, Yu Y (2010). Antisense treatment of IGF-IR enhances chemosensitivity in squamous cell carcinomas of the head and neck. *European Journal of Cancer*.

[73] Hewish M, Chau I, Cunningham D (2009). Insulin-like growth factor 1 receptor targeted therapeutics: novel compounds and novel treatment strategies for cancer medicine. *Recent Patents on Anti-Cancer Drug Discovery*.

[74] Weroha SJ, Haluska P (2008). IGF-1 receptor inhibitors in clinical trials—early lessons. *Journal of Mammary Gland Biology and Neoplasia*.

[75] Sachdev D (2010). Targeting the Type I insulin-like growth factor system for breast cancer therapy. *Current Drug Targets*.

[76] Camirand A, Zakikhani M, Young F, Pollak M (2005). Inhibition of insulin-like growth factor-1 receptor signaling enhances growth-inhibitory and proapoptotic effects of gefitinib (Iressa) in human breast cancer cells. *Breast Cancer Research*.

[77] Jones HE, Goddard L, Gee JMW (2004). Insulin-like growth factor-I receptor signalling and acquired resistance to gefitinib (ZD1839; Iresa) in human breast and prostate cancer cells. *Endocrine-Related Cancer*.

[78] Trusolino L, Comoglio PM (2002). Scatter-factor and semaphorin receptors: cell signalling for invasive growth. *Nature Reviews Cancer*.

[79] Birchmeier C, Birchmeier W, Gherardi E, Vande Woude GF (2003). Met, metastasis, motility and more. *Nature Reviews Molecular Cell Biology*.

[80] Chirgadze DY, Hepple JP, Zhou H, Andrew Byrd R, Blundell TL, Gherardi E (1999). Crystal structure of the NK1 fragment of HGF/SF suggests a novel mode for growth factor dimerization and receptor binding. *Nature Structural Biology*.

[81] Stamos J, Lazarus RA, Yao X, Kirchhofer D, Wiesmann C (2004). Crystal structure of the HGF *β*-chain in complex with the Sema domain of the Met receptor. *The EMBO Journal*.

[82] Sattler M, Salgia R (2009). The MET axis as a therapeutic target. *Update on Cancer Therapeutics*.

[83] Comoglio PM, Giordano S, Trusolino L (2008). Drug development of MET inhibitors: targeting oncogene addiction and expedience. *Nature Reviews Drug Discovery*.

[84] Eder JP, Vande Woude GF, Boerner SA, Lorusso PM (2009). Novel therapeutic inhibitors of the c-Met signaling pathway in cancer. *Clinical Cancer Research*.

[85] Seiwert TY, Jagadeeswaran R, Faoro L (2009). The MET receptor tyrosine kinase is a potential novel therapeutic target for head and neck squamous cell carcinoma. *Cancer Research*.

[86] Cowin AJ, Kallincos N, Hatzirodos N (2001). Hepatocyte growth factor and macrophage-stimulating protein are upregulated during excisional wound repair in rats. *Cell and Tissue Research*.

[87] Di Renzo MF, Olivero M, Martone T (2000). Somatic mutations of the MET oncogene are selected during metastatic spread of human HNSC carcinomas. *Oncogene*.

[88] Cortesina G, Martone T, Galeazzi E (2000). Staging of head and neck squamous cell carcinoma using the MET oncogene product as marker of tumor cells in lymph node metastases. *International Journal of Cancer*.

[89] Tuynman JB, Lagarde SM, Ten Kate FJW, Richel DJ, Van Lanschot JJB (2008). Met expression is an independent prognostic risk factor in patients with oesophageal adenocarcinoma. *British Journal of Cancer*.

[90] Kim C-H, Koh YW, Han JH (2010). C-Met expression as an indicator of survival outcome in patients with oral tongue carcinoma. *Head and Neck*.

[91] Knowles LM, Stabile LP, Egloff AM (2009). HGF and c-Met participate in paracrine tumorigenic pathways in head and neck squamous cell cancer. *Clinical Cancer Research*.

[92] Kakkar T, Ma M, Zhuang Y, Patton A, Hu Z, Mounho B (2007). Pharmacokinetics and safety of a fully human hepatocyte growth factor antibody, AMG 102, in cynomolgus monkeys. *Pharmaceutical Research*.

[93] Gordon MS, Sweeney CS, Mendelson DS (2010). Safety, pharmacokinetics, and pharmacodynamics of AMG 102, a fully human hepatocyte growth factor-neutralizing monoclonal antibody, in a first-in-human study of patients with advanced solid tumors. *Clinical Cancer Research*.

[94] Rosen PJ, Sweeney CJ, Park DJ (2010). A phase Ib study of AMG 102 in combination with bevacizumab or motesanib in patients with advanced solid tumors. *Clinical Cancer Research*.

[95] Jin H, Yang R, Zheng Z (2008). MetMAb, the one-armed 5d5 anti-c-met antibody, inhibits orthotopic pancreatic tumor growth and improves survival. *Cancer Research*.

[96] Martens T, Schmidt NO, Eckerich C (2006). A novel one-armed anti-c-Met antibody inhibits glioblastoma growth in vivo. *Clinical Cancer Research*.

[97] Qian F, Engst S, Yamaguchi K (2009). Inhibition of tumor cell growth, invasion, and metastasis by EXEL-2880 (XL880, GSK1363089), a novel inhibitor of HGF and VEGF receptor tyrosine kinases. *Cancer Research*.

[98] Munshi N, Jeay S, Li Y (2010). ARQ 197, a novel and selective inhibitor of the human c-Met receptor tyrosine kinase with antitumor activity. *Molecular Cancer Therapeutics*.

[99] Bagai R, Fan W, Ma PC (2010). ARQ-197, an oral small-molecule inhibitor of c-met for the treatment of solid tumors. *IDrugs*.

[100] Bigner SH, Humphrey PA, Wong AJ (1990). Characterization of the epidermal growth factor receptor in human glioma cell lines and xenografts. *Cancer Research*.

[101] Moscatello DK, Holgado-Madruga M, Emlet DR, Montgomery RB, Wong AJ (1998). Constitutive activation of phosphatidylinositol 3-kinase by a naturally occurring mutant epidermal growth factor receptor. *The Journal of Biological Chemistry*.

[102] Leeman-Neill RJ, Wheeler SE, Singh SV (2009). Guggulsterone enhances head and neck cancer therapies via inhibition of signal transducer and activator of transcription-3. *Carcinogenesis*.

[103] Wheeler SE, Suzuki S, Thomas SM (2010). Epidermal growth factor receptor variant III mediates head and neck cancer cell invasion via STAT3 activation. *Oncogene*.

[104] Gillison ML (2004). Human papillomavirus-associated head and neck cancer is a distinct epidemiologic, clinical, and molecular entity. *Seminars in Oncology*.

[105] Califano J, van der Riet P, Westra W (1996). Genetic progression model for head and neck cancer: implications for field cancerization. *Cancer Research*.

[106] Richards KL, Zhang B, Baggerly KA (2009). Genome-wide hypomethylation in head and neck cancer is more pronounced in HPV-negative tumors and is associated with genomic instability. *PLoS One*.

[107] Woenckhaus J, Steger K, Werner E (2002). Genomic gain of PIK3CA and increased expression of pI I 0alpha are associated with progression of dysplasia into invasive squamous cell carcinoma. *Journal of Pathology*.

[108] Grandis JR, Drenning SD, Chakraborty A (1998). Requirement of Stat3 but not Stat1 activation for epidermal growth factor receptor-mediated cell growth in vitro. *The Journal of Clinical Investigation*.

[109] Leeman RJ, Lui VWY, Grandis JR (2006). STAT3 as a therapeutic target in head and neck cancer. *Expert Opinion on Biological Therapy*.

[110] Masuda M, Suzui M, Yasumatu R (2002). Constitutive activation of signal transducers and activators of transcription 3 correlates with cyclin D1 overexpression and may provide a novel prognostic marker in head and neck squamous cell carcinoma. *Cancer Research*.

[111] Clark LJ, Edington K, Swan IRC (1993). The absence of Harvey ras mutations during development and progression of squamous cell carcinomas of the head and neck. *British Journal of Cancer*.

[112] Saranath D, Chang SE, Bhoite LT (1991). High frequency mutation in codons 12 and 61 of H-ras oncogene in chewing tobacco-related human oral carcinoma in India. *British Journal of Cancer*.

[113] Engelman JA, Luo JI, Cantley LC (2006). The evolution of phosphatidylinositol 3-kinases as regulators of growth and metabolism. *Nature Reviews Genetics*.

[114] Scarpino S, d’Alena FC, Di Napoli A, Pasquini A, Marzullo A, Ruco LP (2004). Increased expression of Met protein is associated with up-regulation of hypoxia inducible factor-I (HIF-I) in tumour cells in papillary carcinoma of the thyroid. *Journal of Pathology*.

[115] Xu L, Nilsson MB, Saintigny P (2010). Epidermal growth factor receptor regulates MET levels and invasiveness through hypoxia-inducible factor-1*α* in non-small cell lung cancer cells. *Oncogene*.

[116] Pennacchietti S, Michieli P, Galluzzo M, Mazzone M, Giordano S, Comoglio PM (2003). Hypoxia promotes invasive growth by transcriptional activation of the met protooncogene. *Cancer Cell*.

[117] Pugh CW, Ratcliffe PJ (2003). Regulation of angiogenesis by hypoxia: role of the HIF system. *Nature Medicine*.

[118] Hara S, Nakashiro KI, Klosek SK, Ishikawa T, Shintani S, Hamakawa H (2006). Hypoxia enhances c-Met/HGF receptor expression and signaling by activating HIF-1*α* in human salivary gland cancer cells. *Oral Oncology*.

[119] Chakravarti A, Loeffler JS, Dyson NJ (2002). Insulin-like growth factor receptor I mediates resistance to anti-epidermal growth factor receptor therapy in primary human glioblastoma cells through continued activation of phosphoinositide 3-kinase signaling. *Cancer Research*.

